# Supplementary material for: Resolving the full spectrum of human genome variation using Linked-Reads
Source: Genome Res. 2019 Apr;29(4):635–45. doi: 10.1101/gr.234443.118 (PMC6442396; doi:10.1101/gr.234443.118)
Supplement: Supplemental Material [file supp_gr.234443.118_Supplemental_longranger-2.2.zip › longranger-2.2.tar.bz2!/lr2.2-public/lib/go/src/code.google.com/vitess/web/vtctld2/src/app/shared/dialog/dialog.component.html]

#### {{dialogSettings.dialogSubtitle}}

**{{flag.name}} (Required):**

{{flag.description}}

### Command:

{{cmd}}

{{dialogSettings.actionWord}}
Cancel

{{dialogContent.interpolateMessage(dialogSettings.respText)}}

#### Log:

```
{{dialogSettings.logText}}
```

Loading Response...

Dismiss
